# Supine MDS-UPDRS-III Assessment: An Explorative Study

**DOI:** 10.3390/jcm12093108

**Published:** 2023-04-25

**Authors:** Naomi I. Kremer, Annemarie Smid, Stèfan F. Lange, Iara Mateus Marçal, Katalin Tamasi, J. Marc C. van Dijk, Teus van Laar, Gea Drost

**Affiliations:** 1 Department of Neurosurgery, University Medical Center Groningen, University of Groningen, Hanzeplein 1, 9713 GZ Groningen, The Netherlands; 2 Department of Epidemiology, University Medical Center Groningen, University of Groningen, Hanzeplein 1, 9713 GZ Groningen, The Netherlands; 3 Expertise Center Movement Disorders Groningen, University Medical Center Groningen, Hanzeplein 1, 9713 GZ Groningen, The Netherlands; 4 Department of Neurology, University Medical Center Groningen, University of Groningen, Hanzeplein 1, 9713 GZ Groningen, The Netherlands

**Keywords:** MDS-UPDRS, motor section, validation, supine, accelerometry

## Abstract

The Movement Disorder Society Unified Parkinson’s Disease Rating Scale—part III (MDS-UPDRS-III) is designed to be applied in the sitting position. However, to evaluate the clinical effect during stereotactic neurosurgery or to assess bedridden patients with Parkinson’s disease (PD), the MDS-UPDRS-III is often used in a supine position. This explorative study evaluates the agreement of the MDS-UPDRS-III in the sitting and the supine positions. In 23 PD patients, the MDS-UPDRS-III was applied in both positions while accelerometric measurements were performed. Video recordings of the assessments were evaluated by two certified raters. Agreement between the sitting and supine MDS-UPDRS-III was studied using Cohen’s kappa coefficient. Relationships between the MDS-UPDRS-III tremor scores and accelerometric amplitudes were calculated for both positions with linear regression. A fair to substantial agreement was found for MDS-UPDRS-III scores of individual items in the sitting and supine positions, while combining all tests resulted in a substantial agreement. The inter-rater reliability was fair to moderate for both positions. A logarithmic relationship between tremor scores and accelerometric amplitude was revealed for both the sitting and supine positions. Nevertheless, these data are insufficient to fully support the supine application of the MDS-UPDRS-III. Several recommendations are made to address the sensitivity of the scale to inter-rater variability. In conclusion, although an overall substantial agreement between sitting and supine MDS-UPDRS-III is confirmed, its application in the supine position is not endorsed for the whole range of its individual items. Caution is warranted in interpreting the supine MDS-UPDRS-III, pending additional research.

## 1. Introduction

The motor section (part III) of the Movement Disorder Society Unified Parkinson’s Disease Rating Scale (MDS-UPDRS-III) [1] is designed to be applied in the sitting position, but it is routinely used in the supine position to assess bedridden patients with Parkinson’s disease (PD), and to intraoperatively assess the clinical effect of stereotactic neurosurgery [2]. For example, deep brain stimulation (DBS) is a well-known and effective treatment for suppressing debilitating motor symptoms in patients suffering from PD [2]. Correct stereotactic electrode placement is essential for achieving successful treatment outcomes. The intraoperative improvement of motor symptoms during awake stereotactic neurosurgery indicates correct electrode placement [2]. Monitoring these motor symptoms intraoperatively allows for the optimization of electrode position and subsequently the treatment effect, underlining the importance of accurate intraoperative monitoring.

The MDS-UPDRS-III consists of 18 tests: (1) speech assessment, (2) facial expression, (3) rigidity, (4) finger tapping, (5) hand movements, (6) pronation-supination movements of the hands, (7) toe tapping, (8) leg agility, (9) arising from a chair, (10) gait, (11) freezing of gait, (12) postural stability, (13) posture, (14) global spontaneity of movement (body bradykinesia), (15) postural tremor of the hands, (16) kinetic tremor of the hands, (17) rest tremor amplitude, and (18) rest tremor constancy [1]. Test 3 is performed for the neck, right upper extremities (RUE), left upper extremities (LUE), right lower extremities (RLE), and left lower extremities (LLE). Tests 4–8 and 15–16 are performed on both the left and right side, and test 17 is performed for all extremities and the lip/jaw, resulting in 33 tasks in total [1].

During surgery, MDS-UPDRS-III testing is performed in the supine position, since patients are situated on the operating table with the head fixated in a stereotactic frame. Several studies have used the MDS-UPDRS-III in the supine position [2,3,4,5,6], even though the MDS-UPDRS-III was originally validated in the sitting position [7]. Therefore, the primary aim of this prospective study is to investigate the agreement between MDS-UPDRS-III assessments in sitting and supine position, in order to endorse the supine application of the MDS-UPDRS-III. In addition, this study aims to investigate the inter-rater reliability and the relation between the MDS-UPDRS-III and accelerometric measurements in the sitting versus supine positions.

## 2. Materials and Methods

### 2.1. Participants

Adult PD patients were eligible if they were diagnosed by UK Brain Bank criteria [8] and were Hoehn and Yahr stage I–IV [9]. Exclusion criteria were any form of musculoskeletal system disorders, physical disabilities, and recent alcohol/drug abuse. Exemption from the act on research involving human subjects was granted by the local research ethical board. This study was conducted in compliance with the Helsinki Declaration for research on human beings. Written informed consent was obtained from each participant.

### 2.2. Materials

Two wired tri-axial accelerometers (MMA8452Q tri-Axis, Freescale Semiconductor, Inc., Austin, TX, USA) with a ±2 g range and a 200 Hz sampling rate were used, as previously described by Smid et al. [3]. Accelerometry data were recorded with LabVIEW v. 2014 (National Instruments, Austin, TX, USA).

### 2.3. Measurements

Participants were invited to the outpatient clinic to undergo MDS-UDPRS-III assessments [1], which took on average 39 min (SD ± 16.9) to complete. Participants were instructed to use their usual Parkinsonian medication; time of last intake was noted. If applicable, the DBS device was turned off during the measurements. Of the 33 MDS-UDPRS-III tasks, items 1–8 and 14–18 were first performed in sitting position and subsequently items 1, 2, 4–6, and 14–18 in supine position. In the latter position, MDS-UDPRS-III item 3 could not be performed as there was no neurologist available to perform the rigidity assessment. Items 7 and 8 could not be performed in the supine position. MDS-UDPRS-III items 9–13 were performed once, as these were not possible in sitting or supine positions, in order to calculate the total MDS-UPDRS-III score. All tasks were recorded on video. Accelerometry data (with sensors on both index fingers) were recorded for upper extremity tremor (items 15 and 17), as described previously [3].

### 2.4. Video Ratings

Two certified raters, blinded for patient characteristics, independently assessed MDS-UPDRS-III from video recordings. Recordings could be rewatched if necessary. To prevent bias, scoring of sitting and supine tasks of various participants was random.

### 2.5. Accelerometry Analysis

Signal analysis was performed in MATLAB v. 2021a (MathWorks, Natick, MA, USA). Pre-processing of tri-axial acceleration data was performed as described by Smid et al. [3]. Accelerometric data measured during the upper extremity tremor tasks (items 15 and 17) were used to assess postural and rest tremor amplitude. The amplitude was calculated by determining the mean of all peaks in the twice-integrated absolute displacement vector, multiplied by two.

### 2.6. Statistical Analysis

Statistical analysis was performed in IBM SPSS v. 28 (International Business Machines Corporation, New York, NY, USA).

#### 2.6.1. Primary Endpoint

Agreement between sitting and supine MDS-UDPRS-III scores was evaluated with Cohen’s weighted kappa coefficient (κ). MDS-UDPRS-III items 1–2, 4–6, and 14–18 were used to calculate agreement between both positions. Item scores of both raters were averaged and rounded before calculating agreement. An agreement of κ > 0.6 between sitting versus supine was considered acceptable [10,11].

#### 2.6.2. Secondary Endpoints

For 28 of 33 MDS-UDPRS-III tasks, Cohen’s weighted kappa coefficient (κ) was used to assess inter-rater reliability between the 2 MDS-UDPRS-III raters. The five rigidity tasks were excluded as these were only available in the sitting position.

Contrast coding of MDS-UDPRS-III tremor scores was defined to be orthogonal polynomial, of which the first contrast coefficient has a linear trend, to assess linearity between MDS-UPDRS tremor scores and the logarithm (log10) of the accelerometric tremor amplitude [3,12], assessed separately in sitting and supine positions. MDS-UPDRS tremor scores of the two raters were averaged and rounded per measurement.

## 3. Results

Twenty-three patients (four women; 61.5 ± 9.1 years) with PD were included. The average disease duration was 8.0 ± 5.4 years. The total MDS-UDPRS-III score was 37 ± 15. The left hemibody was predominantly affected in 12 patients, while in 9 patients the right side was more affected. All measurements were performed with the patient in ON state. In 19 patients, the median interval (interquartile range) since last medication at the start of the measurements was 105 (140) min. In 13 patients, a DBS device was present. At the time of the assessment, these participants had been treated for 20.6 ± 14.4 months on average (±SD).

### 3.1. Primary Endpoint: MDS-UPDRS-III Agreement in Sitting and Supine Positions

**Figure 1:**
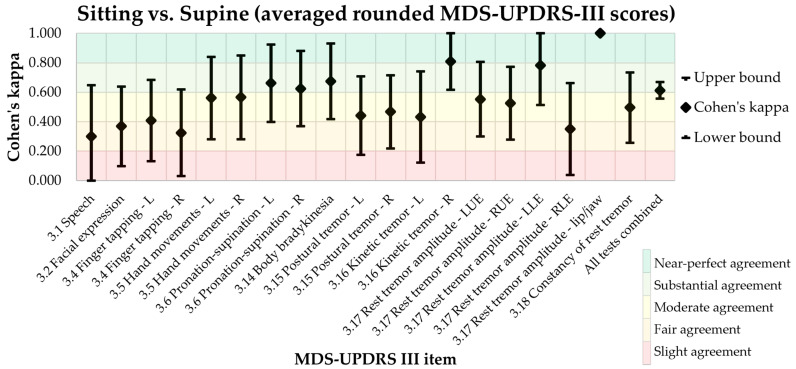
Cohen’s kappa for sitting and supine MDS-UDPRS-III scores. L: Left side. R: Right side. LUE: Left upper extremities. RUE: Right upper extremities. LLE: Left lower extremities. RLE: Right lower extremities.

Cohen’s κ coefficient between sitting and supine MDS-UDPRS-III is shown in Table 1 and Figure 1. Aside from the outliers, a fair to substantial agreement was shown for the individual items, while combining all tests resulted in a substantial agreement for MDS-UDPRS-III scores in the sitting and supine positions [10,11]. Overall agreement (all tests combined) was beyond the acceptance level of 0.6 (κ = 0.613). However, the agreement between sitting and supine position was below the 0.6 threshold for several items (Table 1).

Cohen’s κ results showed a perfect agreement for lip/jaw tremor and a near-perfect agreement for kinetic tremor of the right hand. A substantial agreement was shown for pronation/supination, body bradykinesia, and rest tremor of the left lower extremity, while a moderate agreement was shown for finger tapping of the left hand, hand movements, postural tremor, kinetic tremor of the left hand, rest tremor of the left and right upper extremities, and constancy of rest tremor. A fair agreement was shown for speech, facial expression, finger tapping of the right hand, and right lower extremity rest tremor [10,11].

### 3.2. Secondary Endpoints

#### 3.2.1. Inter-Rater Reliability

The inter-rater reliability for sitting and supine MDS-UDPRS-III was calculated (see Table 2 and Table 3). For the sitting position, the Cohen’s κ coefficient ranged from −0.20 to 1.00, and for the supine position, from −0.14 to 1.00 in. Aside from the outliers, the inter-rater reliability was fair to moderate in both positions [10,11].

#### 3.2.2. The Relationship between MDS-UPDRS-III Scores and Accelerometric Amplitude

**Figure 2:**
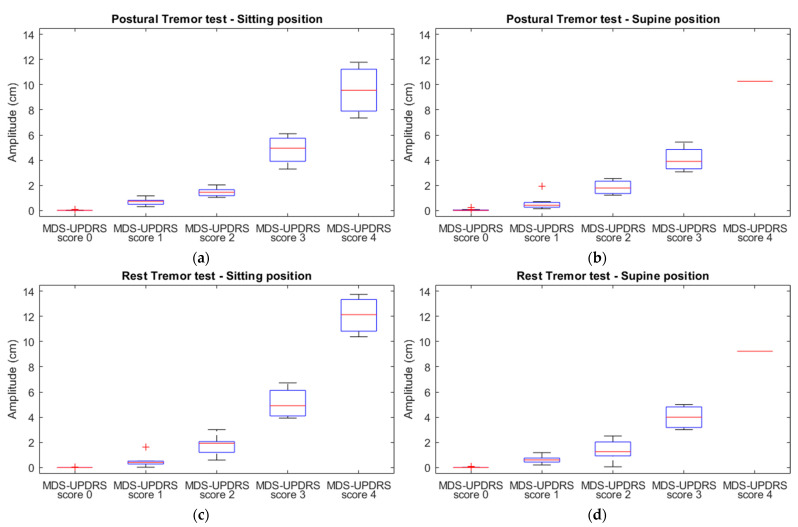
Boxplots of the tremor amplitude of the sitting postural (**a**), supine postural (**b**), sitting rest (**c**), and supine rest (**d**) tremor tests (per MDS-UPDRS score).

In Figure 2, the accelerometric amplitude of resting and postural tremor tests in the sitting and supine positions are plotted against the given averaged MDS-UPDRS-III tremor scores. An accelerating increase in amplitude with increasing MDS-UPDRS-III score is noted in all subfigures. Hence, the log-transformed accelerometric tremor amplitudes of sitting and supine postural and rest tremor were regressed on the corresponding MDS-UPDRS-III tremor scores. There was strong evidence against the null hypothesis that the log-transformed amplitude was not linearly related to the MDS-UPDRS-III scores (*p* < 0.001) for both tremor tests and in both positions. The amplitude tended to increase as a logarithmic function of MDS-UDPRS-III scores (R^2^ > 0.712) for all tests (Table 4).

## 4. Discussion

This study revealed a fair to substantial agreement for sitting and supine MDS-UPDRS-III scores. The inter-rater reliability was fair to moderate in both positions. The relationship between tremor amplitude and clinical tremor score in sitting position was shown to be similar to that in the supine position for both rest and postural tremors. Trends were consistent with the logarithmic relationship between tremor amplitude and clinical tremor score reported in previous studies [3,12].

In this explorative study, an acceptable overall agreement (all tests combined) between the sitting and supine position was found. However, several sub-items showed an agreement below the set acceptance level. Consequently, the supine use of the MDS-UPDRS-III is discouraged based on these data. Additional research is warranted and perhaps an alternative version of the MDS-UPDRS-III should be designed, preferably by the expert panel of the MDS, for supine assessment of the items for which disagreement was shown in our study.

When assessing all MDS-UDPRS-III items separately, Cohen’s κ coefficient between the scores in sitting and supine position had a wide range. This is explained by the small sample size. All MDS-UDPRS-III tests combined produced a substantial agreement with a relatively narrow 95% confidence interval. It was notable that there was no clear subgroup of items that consistently showed substantial agreement, or no agreement at all. In some patients the ratings for speech were higher in supine position than in sitting position. This could be due to gravitational force, which can influence speaking while reclining [13]. In the operative setting, decreased oral fluid intake and higher stress level may cause a dry mouth, worsening speech. The perfect agreement found for lip/jaw tremor (Table 1, Table 2 and Table 3) is explained by the fact that only 1 of the 23 participants had this symptom. Some of the lower agreements found between the sitting and supine position could also be accounted for by the relatively low inter-rater reliability found in this study, e.g., for the speech, hand movements and pronation/supination items. These findings are in line with those of previous studies, which have shown that the MDS-UPDRS-III within-patient reliability consists of a substantial amount of error variance, especially for the bradykinetic items [14,15,16,17,18]. The poor inter-rater reliability found for item 3.1 (Table 2 and Table 3) could be explained by the speech criteria being open to interpretation, as it can differ between raters whether the words or sentences spoken by the patient are easy or poorly understood by the rater. This might result in different raters giving different ratings (e.g., with a difference of one point) to the same speech assessment. The disagreement found for item 3.11 (Table 2) occurred because none of the participants suffered from freezing, so the inter-rater reliability could not be properly calculated for that item.

### 4.1. Limitations

All measurements were initially performed in the sitting position and subsequently in the supine position. This might have introduced order effects, such as the influence of medication status and fatigue on the results, although measurements were performed in a short interval. Rigidity was not assessed in the supine position in this study due to logistic reasons. Additionally, facial expression could not be assessed properly during supine assessments, as the camera was positioned at the participant’s feet, making it difficult to observe whether the lips were parted and explaining the lower agreement found for this item. Furthermore, the study uses a convenience sample—no formal calculation was performed as test variability could not be estimated.

### 4.2. Recommendations

In this study, it was shown that lower extremity tremor assessment can differ in supine versus sitting position. Since the legs are supported by the bed in supine position, this might have caused the amplitude of a lower extremity tremor to be suppressed. Moreover, since the legs are often positioned under blankets during surgery, their tremor amplitude may be suppressed even more. Therefore, it is recommended to be aware of the position of the extremities during supine assessment of tremor.

Some MDS-UDPRS-III items (e.g., rigidity and tremor amplitude) are described in such a way that inter-rater variability is limited, as its criteria are less subject to interpretation. These items showed a higher inter-rater reliability in this study. Other items (e.g., the bradykinetic items) are more susceptible to inter-rater variability, since criteria as “slight”, “mild”, and “moderate” are open to interpretation. It can also differ per rater whether “slowing” of the movement during the task is rated, versus general “slowness” of movement during the whole task. Most importantly, the correct scoring of these items depends on the instructions given to the patient, which will influence how the task is performed. Criteria should be defined more clearly to minimize inter-rater variability.

### 4.3. Future Perspectives

Although many studies already applied the MDS-UPDRS-III supinely [2,3,4,5,6], its use has not been validated in this position. In this explorative study, the agreement between MDS-UPDRS-III application in the sitting and supine positions was evaluated, and demonstrated that sitting and supine assessments may differ. However, in order to properly validate the supine application of the MDS-UPDRS-III, further research is indispensable. Additional experiments should be performed to determine the reproducibility of MDS-UPDRS-III items, in order to test variability between sitting and supine assessments to be assigned to the position in which measurements were performed. Rigidity assessments should be performed in the supine position to include the results of this item in the validation analysis. Additionally, order effects should be prevented by proper counterbalancing. The use of several video cameras from different angles could prevent difficulties with properly observing the participant. The sample size of future studies could be calculated based on the test variability of the current study. Therefore, a large-scale validation study is proposed, in which reproducibility analysis, order effects, and supine rigidity items are considered. As such, supine MDS-UPDRS-III application will be properly validated, or, in the case of unconvincing results, an alternative version of the MDS-UPDRS-III could be designed for supine use.

## 5. Conclusions

Although an overall substantial agreement between the MDS-UPDRS-III in the sitting and supine position is confirmed, its application in the supine position is not endorsed for the whole range of its individual items. In general, the MDS-UPDRS-III is found to be sensitive to inter-rater variability, for which recommendations are formulated. At present, in anticipation of additional research, caution is warranted in interpreting the supine MDS-UPDRS-III. In future studies, the test variability of the current study can be used to determine the sample size.

## Figures and Tables

**Table 1:** Cohen’s kappa results: sitting versus supine (averaged rounded MDS-UPDRS scores).

MDS-UDPRS-III Test	Site	Cohen’s Kappa	95% CI	*p*
3.1 Speech		0.300	−0.049, 0.649	0.073
3.2 Facial expression		0.369	0.099, 0.639	<0.001
3.4 Finger tapping	Left	0.407	0.131, 0.683	<0.001
	Right	0.325	0.031, 0.619	0.007
3.5 Hand movements	Left	0.561	0.281, 0.841	<0.001
	Right	0.566	0.282, 0.850	<0.001
3.6 Pronation/supination	Left	0.662	0.399, 0.925	<0.001
	Right	0.625	0.370, 0.880	<0.001
3.14 Body bradykinesia		0.674	0.417, 0.931	<0.001
3.15 Postural tremor	Left	0.441	0.174, 0.708	<0.001
	Right	0.467	0.218, 0.716	<0.001
3.16 Kinetic tremor	Left	0.432	0.122, 0.742	<0.001
	Right	0.810	0.616, 1.004	<0.001
3.17 Rest tremor amplitude	LUE	0.553	0.300, 0.806	<0.001
	RUE	0.525	0.278, 0.772	<0.001
	LLE	0.782	0.513, 1.051	<0.001
	RLE	0.350	0.038, 0.662	0.030
	Lip/jaw	1.000	1.000, 1.000	<0.001
3.18 Constancy of rest tremor		0.496	0.257, 0.735	<0.001
All tests combined		0.613	0.556, 0.670	<0.001

**Table 2:** Cohen’s kappa results: Inter-rater sitting position rater 1 versus rater 2.

MDS-UDPRS-III Test	Site	Cohen’s Kappa	95% CI	*p*
3.1 Speech		−0.196	−0.435, 0.043	0.132
3.2 Facial expression		0.228	−0.021, 0.477	0.029
3.4 Finger tapping	Left	0.378	0.119, 0.637	<0.001
	Right	0.346	0.091, 0.601	0.002
3.5 Hand movements	Left	0.051	−0.169, 0.271	0.598
	Right	−0.040	−0.297, 0.217	0.744
3.6 Pronation-supination	Left	0.111	−0.093, 0.315	0.160
	Right	0.122	−0.117, 0.361	0.216
3.7 Toe tapping	Left	0.501	0.242, 0.760	<0.001
	Right	0.141	−0.071, 0.353	0.183
3.8 Leg agility	Left	0.351	0.084, 0.618	0.002
	Right	0.237	−0.004, 0.478	0.024
3.9 Arising from chair		0.704	0.388, 1.020	<0.001
3.10 Gait		0.473	0.154, 0.792	0.005
3.11 Freezing of gait		0.000	0.000, 0.000	<0.001
3.12 Postural stability		0.629	0.384, 0.874	<0.001
3.13 Posture		0.385	0.124, 0.646	<0.001
3.14 Body bradykinesia		0.075	−0.168, 0.318	0.462
3.15 Postural tremor	Left	0.550	0.291, 0.809	<0.001
	Right	0.786	0.574, 0.998	<0.001
3.16 Kinetic tremor	Left	0.398	0.071, 0.725	0.003
	Right	0.339	0.092, 0.586	0.009
3.17 Rest tremor amplitude	LUE	0.574	0.343, 0.805	<0.001
	RUE	0.742	0.526, 0.958	<0.001
	LLE	0.429	0.100, 0.758	0.005
	RLE	−0.045	−0.112, 0.022	0.766
	Lip/jaw	1.000	1.000, 1.000	<0.001
3.18 Constancy of rest tremor		0.468	0.239, 0.697	<0.001
All tests combined		0.431	0.382, 0.480	<0.001

**Table 3:** Cohen’s kappa results: Inter-rater supine position rater 1 versus rater 2.

MDS-UDPRS-III Test	Site	Cohen’s Kappa	95% CI	*p*
3.1 Speech		−0.135	−0.284, 0.014	0.104
3.2 Facial expression		0.140	−0.081, 0.361	0.140
3.4 Finger tapping	Left	0.371	0.102, 0.640	<0.001
	Right	0.303	0.031, 0.575	0.008
3.5 Hand movements	Left	0.061	−0.172, 0.294	0.547
	Right	0.168	−0.079, 0.415	0.138
3.6 Pronation-supination	Left	0.212	0.000, 0.424	0.011
	Right	−0.005	−0.164, 0.154	0.954
3.14 Body bradykinesia		0.250	−0.013, 0.513	0.033
3.15 Postural tremor	Left	0.512	0.242, 0.782	<0.001
	Right	0.717	0.462, 0.972	<0.001
3.16 Kinetic tremor	Left	0.129	−0.138, 0.396	0.198
	Right	0.457	0.218, 0.696	<0.001
3.17 Rest tremor amplitude	LUE	0.540	0.268, 0.812	<0.001
	RUE	0.509	0.256, 0.762	<0.001
	LLE	0.416	0.153, 0.679	0.003
	RLE	0.361	0.057, 0.665	0.013
	Lip/jaw	1.000	1.000, 1.000	<0.001
3.18 Constancy of rest tremor		0.604	0.392, 0.816	<0.001
All tests combined		0.385	0.326, 0.444	<0.001

**Table 4:** Regression analysis results.

MDS-UPDRS-III Test	Position	R	R^2^ *	Coefficient **	95% CI	*p*
Postural tremor	Sitting	0.900	0.811	2.662	2.271, 3.052	<0.001
	Supine	0.862	0.743	2.873	2.360, 3.387	<0.001
Rest tremor	Sitting	0.905	0.818	3.604	3.088, 4.121	<0.001
	Supine	0.844	0.712	4.213	3.399, 5.027	<0.001

* Coefficient of determination; ** Contrast coefficient testing for linear trend.

## Data Availability

The data may be available upon reasonable request.

## References

[1] Goetz C.G., Fahn S., Martinez-Martin P., Poewe W., Sampaio C., Stebbins G.T., Stern M.B., Tilley B.C., Dodel R., Dubois B. The MDS-Sponsored Revision of the Unified Parkinson’s Disease Rating Scale. www.movementdisorders.org.

[2] Lange S.F., Kremer N.I., van Laar T., Lange F., Steendam-Oldekamp T.E., Oterdoom D.L.M., Absalom A.R., van Dijk J.M.C., Drost G. (2021). The Intraoperative Microlesion Effect Positively Correlates with the Short-Term Clinical Effect of Deep Brain Stimulation in Parkinson’s Disease. Neuromodulation.

[3] Smid A., Elting J.W.J., van Dijk J.M.C., Otten B., Oterdoom D.L.M., Tamasi K., Heida T., van Laar T., Drost G. (2022). Intraoperative Quantification of MDS-UPDRS Tremor Measurements Using 3D Accelerometry: A Pilot Study. J. Clin. Med..

[4] Krauss P., Oertel M.F., Baumann-Vogel H., Imbach L., Baumann C.R., Sarnthein J., Regli L., Stieglitz L.H. (2021). Intraoperative Neurophysiologic Assessment in Deep Brain Stimulation Surgery and Its Impact on Lead Placement. J. Neurol. Surg. A Cent. Eur. Neurosurg..

[5] Wu J., Yu N., Yu Y., Li H., Wu F., Yang Y., Lin J., Han J., Liang S. (2021). Intraoperative Quantitative Measurements for Bradykinesia Evaluation during Deep Brain Stimulation Surgery Using Leap Motion Controller: A Pilot Study. Parkinsons Dis..

[6] Yu N., Yu Y., Lin J., Yang Y., Wu J., Liang S., Wu J., Han J. (2022). A Non-Contact System for Intraoperative Quantitative Assessment of Bradykinesia in Deep Brain Stimulation Surgery. Comput. Methods Programs Biomed..

[7] Goetz C.G., Tilley B.C., Shaftman S.R., Stebbins G.T., Fahn S., Martinez-Martin P., Poewe W., Sampaio C., Stern M.B., Dodel R. (2008). Movement Disorder Society-Sponsored Revision of the Unified Parkinson’s Disease Rating Scale (MDS-UPDRS): Scale Presentation and Clinimetric Testing Results. Mov. Disord..

[8] Hughes A.J., Daniel S.E., Kilford L., Lees A.J. (1992). Accuracy of Clinical Diagnosis of Idiopathic Parkinson’s Disease: A Clinico-Pathological Study of 100 Cases. J. Neurol. Neurosurg. Psychiatry.

[9] Hoehn M.M., Yahr M.D. (1967). Parkinsonism: Onset, Progression, and Mortality. Neurology.

[10] McHugh M. (2012). Interrater Reliability—The Kappa Statistic. Biochem. Med..

[11] Ranganathan P., Pramesh C., Aggarwal R. (2017). Common Pitfalls in Statistical Analysis: Measures of Agreement. Perspect. Clin. Res..

[12] Elble R.J., Pullman S.L., Matsumoto J.Y., Raethjen J., Deuschl G., Tintner R. (2006). Tremor Amplitude Is Logarithmically Related to 4- and 5-Point Tremor Rating Scales. Brain.

[13] Shiller D.M., Ostry D.J., Gribble P.L. (1999). Effects of gravitational load on jaw movements in speech. J. Neurosci..

[14] Post B., Merkus M.P., de Bie R.M.A., de Haan R.J., Speelman J.D. (2005). Unified Parkinson’s Disease Rating Scale Motor Examination: Are Ratings of Nurses, Residents in Neurology, and Movement Disorders Specialists Interchangeable?. Mov. Disord..

[15] Yang K., Xiong W.X., Liu F.T., Sun Y.M., Luo S., Ding Z.T., Wu J.J., Wang J. (2016). Objective and Quantitative Assessment of Motor Function in Parkinson’s Disease-from the Perspective of Practical Applications. Ann. Transl. Med..

[16] Evers L.J.W., Krijthe J.H., Meinders M.J., Bloem B.R., Heskes T.M. (2019). Measuring Parkinson’s Disease over Time: The Real-World within-Subject Reliability of the MDS-UPDRS. Mov. Disord..

[17] Rovini E., Maremmani C., Cavallo F. (2017). How Wearable Sensors Can Support Parkinson’s Disease Diagnosis and Treatment: A Systematic Review. Front. Neurosci..

[18] Lu R., Xu Y., Li X., Fan Y., Zeng W., Tan Y., Ren K., Chen W., Cao X. (2020). Evaluation of Wearable Sensor Devices in Parkinson’s Disease: A Review of Current Status and Future Prospects. Parkinsons Dis..

